# Lung cancer mortality in towns near paper, pulp and board industries in Spain: a point source pollution study

**DOI:** 10.1186/1471-2458-8-288

**Published:** 2008-08-14

**Authors:** Susana Monge-Corella, Javier García-Pérez, Nuria Aragonés, Marina Pollán, Beatriz Pérez-Gómez, Gonzalo López-Abente

**Affiliations:** 1 Cancer and Environmental Epidemiology Area, National Centre for Epidemiology, Carlos III Institute of Health, Madrid, Spain; 2 CIBER Epidemiología y Salud Pública (CIBERESP), Madrid, Spain

## Abstract

**Background:**

This study sought to ascertain whether there might be excess lung cancer mortality among the population residing in the vicinity of Spanish paper and board industries which report their emissions to the European Pollutant Emission Register (EPER).

**Methods:**

This was an ecological study that modelled the Standardised Mortality Ratio (SMR) for lung cancer in 8073 Spanish towns over the period 1994–2003. Population exposure to industrial pollution was estimated on the basis of distance from town of residence to pollution source. An exploratory, near-versus-far analysis was conducted, using mixed Poisson regression models and an analysis of the effect of municipal proximity within a 50-kilometre radius of each of the 18 installations.

**Results:**

Results varied for the different facilities. In two instances there was an increasing mortality gradient with proximity to the installation, though this was exclusively observed among men.

**Conclusion:**

The study of cancer mortality in areas surrounding pollutant foci is a useful tool for environmental surveillance, and serves to highlight areas of interest susceptible to being investigated by ad hoc studies. Despite present limitations, recognition is therefore due to the advance represented by publication of the EPER and the study of pollutant foci.

## Background

Lung cancer is the leading cause of cancer-related death among men in Spain, giving rise to 16,614 deaths in 2005, 27.4% of all male cancer-related deaths. In this same year there were 2,459 deaths among women, accounting for 7% of total female deaths and ranking lung cancer as the third leading cause of cancer-related death after breast and colon cancer [1]. The male:female ratio is 7:1. Owing to its frequency and impact, this tumour is regarded as a serious public health problem. Although the male lung-cancer mortality trend has declined in recent years, in women the trend has been rising, particularly from 1990 onwards, and is currently increasing by 2.4% per annum [2]. Despite diagnostic and therapeutic advances, the disease continues to be highly lethal, with only 12.2% of patients surviving to five years after diagnosis [3].

Lung cancer displays marked geographic and temporal variability, which corresponds to the diversity and different distribution of its risk factors. It is estimated that use of tobacco, the principal aetiological agent, attributed to the development of 80%–90% and 55%–80% of cases among men and women respectively [4]. Occupational exposure to different substances, such as arsenic, asbestos, polycyclic aromatic hydrocarbons (PAH), chrome and nickel, can be assumed to be related to 9%–15% of cases [5]. Other acknowledged risk factors are exposure to radon [6], air pollution [7-9]. and a lower intake of fresh fruit and vegetables [5]. Insofar as air pollution is concerned, a leading role is attributed to industrial emissions [10-12], prominent among which are those released by the paper and board sector [13-15].

This sector has been the subject of a number of occupational health studies, inasmuch as its workers are exposed to a range of toxic agents [16] which have been linked to lung cancer [17-20]. In workers of the paper and board industries a statistically significant excess of risk has been found for lung cancer [21-23]. and other tumour sites, such as stomach, breast, ovary, and prostate, as well as mesotheliomas, tumours of the nervous system, haematological tumours, sarcomas and melanomas [24-32]. In the case of lung cancer, excess risks among workers at paper, pulp and board plants have been related to exposure to: sulphur gases and airborne organochlorinated compound mixtures [21,23,33]; asbestos [16,34]; and dust wood [35] and inorganic dust pollutants [36].

Given that some of the pollutants produced by these industries -such as SO_2_, NO_2 _and PM_10 _(particles with a diameter of up to 10 μm)- constitute this sector's usual emissions to the environment [37-39], it would be of interest to assess whether this type of industry might pose a risk to the surrounding population. Indeed, excess risk of lung cancer has been described among the population exposed to greater environmental concentrations of these three substances [8-10,40].

The availability of a new and valuable resource for monitoring industrial pollution in Spain, i.e., the European Pollutant Emission Register (EPER), enables possible related geographic mortality patterns to be ascertained [39,41]. The legal framework for this is provided by Directive 96/61/EC, which envisages the reporting of emissions of 50 pollutant substances in cases where these exceed designated thresholds set by the European Commission. Initially, reporting of emissions was voluntary and then became mandatory as from 2007.

The aim of this study was to ascertain whether there might be excess lung cancer mortality among the population residing in the vicinity of Spanish paper, pulp and board industries which report their emissions to the European Pollutant Emission Register in Spain (EPER-Spain) [42].

## Methods

This was an ecological study that modelled the Standardised Mortality Ratio (SMR) for lung cancer in 8073 Spanish towns over the period 1994–2003.

To calculate the SMRs, we used municipal broncho-pulmonary cancer mortality data -codes 162 (International Classification of Diseases/ICD-9) and C33 and C34 (ICD-10)- drawn from individual death entries over the study period furnished by the National Statistics Institute (*Instituto Nacional de Estadística *– *INE*). Expected cases were calculated by taking the specific rates for Spain as a whole by age group (18 groups: 0–4, 5–9,..., 80–84, 85 and over), sex and five-year period (1994–1998 and 1999–2003), and multiplying these by person-years for each town, broken down by the same age, sex and period strata. For the computation of person-years, the two study quinquennia were considered, with the data corresponding to the 1996 municipal roll and 2001 census taken as the respective population estimators. SMRs were calculated as the ratio of observed to expected deaths, and exact methods were used to establish the 95% confidence intervals (95%CI).

Population exposure to industrial pollution was estimated on the basis of the distance from the town of residence to the pollution source. The distance from each town to the industries considered was calculated by reference to municipal centroids (i.e., the reference point in the largest population centre). Data relating to industries in the paper, pulp and board sector were retrieved from the publicly accessible Internet database of the EPER-Spain [42], by selecting information on Integrated Pollution Prevention and Control (IPPC) categories 6.1.a (manufacture of pulp), 6.1.b (manufacture of paper and board exceeding 20 tonnes/day) and 6.2 (production and treatment of cellulose exceeding 20 tonnes/day). The study was restricted to industries that had reported emissions to air in 2001. Information was obtained on 18 industrial complexes (see Table 1), along with the co-ordinates of their location. The latter were then entered into the Farm Plot Geographic Information System (*Sistema de Información Geográfica de Parcelas Agrícolas*) [43] for verification by reference to orthophotos (digitalised aerial images), and were corrected where necessary [44].

**Table 1:** Paper, pulp and board industries that reported emitting pollutant substances to air in Spain in 2001.

**Industry EPER code**	**Province**	**Sector**	Volume production*	**No. employees**	**NO_2_**	**SO_2_**	**PM_10_**	**CO**	**NMVOC**	**CO_2_**
1599	Pontevedra	Pulp	325000	NA	X	X	X			
1600	Huelva	Pulp	351705	324	X	X	X	X	X	X
1629	Madrid	Paper and board	165000	240	X					
1767	Navarre	Paper and board	121000	NA	X	X				X
2402	Tarragona	Cellulose	10120	112		X				
2761	Zaragoza	Paper and board	450000	285						X
2762	Zaragoza	Paper and board	700000	236	X					X
3067	Zaragoza	Pulp	180000	NA	X		X	X	X	X
3070	Girona	Paper and board	115000	325	X					
3071	Cadiz	Paper and board	49115	NA		X				
3378	Asturias	Pulp	290000	289	X	X	X		X	X
3390	Burgos	Pulp	140000	170	X		X			
3408	Jaen	Paper and board	136	114		X				
3548	Cantabria	Cellulose	66000	NA		X				
3648	Gipuzkoa	Pulp	197294	288	X	X	X	X	X	X
3649	Gipuzkoa	Paper and board	48000	91	X					
3695	Bizkaia	Pulp	114011	214	X	X	X	X	X	
3730	Bizkaia	Pulp	69664	150	X				X	

*In tonnes. NMVOC = non-methane volatile organic compounds. X = pollutants for which emissions exceeding the threshold were reported. NA = data not available. Source: EPER-Spain.

The exposure variable was coded as a "dummy" with the following 3 levels: a) exposed group, namely, towns having their centroid at a distance of less than 2 kilometres from a paper, pulp and board industry; b) intermediate group, namely, towns having their centroid at a distance of less than 2 kilometres from any air polluting industry other than paper, pulp and board; and, c) unexposed group, namely, towns having no EPER-registered industry within a 2-kilometre radius of the municipal centroid (reference level).

Relative risks (RRs) and their 95% confidence intervals were estimated on the basis of a Poisson regression model [45], with the number of expected cases used as offset. To prevent any possible classification errors deriving from the inclusion of large towns, a second model was constructed, eliminating the 626 towns which had over 10,000 inhabitants. Estimated RRs were adjusted for a number of socio-demographic variables, chosen for their availability and potential explanatory ability vis-à-vis certain geographic mortality patterns. These variables were: population size; proportion of illiterates; proportion of persons engaged in farming; percentage of unemployed persons; average number of persons per dwelling, as shown by the 1991 census; and mean income as reported by the Spanish Market Yearbook (*Anuario del Mercado Español*) [46]. Lastly, mixed models were fitted [47], including province as a random-effects term. This enabled extra-Poisson dispersion to be taken into account and unexposed towns belonging to the same geographic setting to be considered as the reference level in each case, something that is justified by the pronounced geographic differences observed in lung cancer mortality [48,49].

Installations' characteristics vary and emission histories (years of operation, number of workers, production volume, emission type and amount) thus tend to be unique to each. In a final phase, therefore, industries in the sector were studied separately (save for those that did not have neighbouring towns), with analyses being confined to the area lying within a radius of 50 km of each so as to have a local comparison group. The risk gradient in the vicinity of each facility was likewise studied, with distance as an explanatory variable, categorised by means of concentric bands of equal radius (< 5 km, 5–10, 10–15, 15–20, 20–50 km) chosen with the objective of have towns in most of the bands for the industries of the sector. The 20–50 km group served as reference. The absence of extra-Poisson dispersion in these models was confirmed by means of Dean's test [50].

## Results

Table 1 shows data on the 18 EPER-registered paper, pulp and board industries, along with the air-pollution profiles reported by each. 11 of those industrial sites had neighbouring towns and where therefore considered in the analysis.

Shown in Table 2 is the number of observed and expected cases, and the SMRs for the 13 towns (3 with over 10,000 inhabitants) having some such installation at a distance of less than 2 km. Most of the effect estimators are close to unity and the only mortality statistically significant excess risk is the result for Zaragoza in men (SMR 1.112; 1.070–1.155).

**Table 2:** Towns less than 2 km from paper and board industries.

				**Total**		**Men**		**Women**	
				
**Town**	**Province**	**Inhab**	**Industry EPER code**	**Obs Exp**	**SMR [95% CI]**	**Obs Exp**	**SMR [95% CI]**	**Obs Exp**	**SMR [95% CI]**
**Sarria de Ter**	Girona	3566	3070	12 16.24	0.739 [0.382–1.291]	11 14.67	0.750 [0.374–1.342]	1 1.57	0.637 [0.016–3.551]
**Aduna**	Gipuzkoa	309	3649	0 1.52	-	0 1.38	-	0 0.13	-
**Zizurkil**	Gipuzkoa	2684	3649	10 11.2	0.893 [0.428–1.641]	8 10.19	0.785 [0.339–1.547]	2 1.01	1.974 [0.239–7.132]
**Hernani**	Gipuzkoa	17490	3648	70 75.55	0.926 [0.722–1.171]	60 67.95	0.883 [0.674–1.137]	10 7.60	1.315 [0.631–2.418]
**Villabona**	Gipuzkoa	5368	3648	19 20.50	0.927 [0.558–1.448]	18 18.47	0.974 [0.578–1.54]	1 2.02	0.494 [0.013–2.754]
**S. Juan del Puerto**	Huelva	5663	1600	21 20.17	1.041 [0.644–1.591]	20 18.21	1.098 [0.671–1.696]	1 1.96	0.511 [0.013–2.846]
**Mengibar**	Jaén	8170	3408	25 29.53	0.846 [0.548–1.250]	25 26.68	0.937 [0.606–1.383]	0 2.85	-
**Sangüesa**	Navarre	4464	1767	20 23.99	0.834 [0.509–1.287]	17 21.67	0.784 [0.457–1.256]	3 2.32	1.293 [0.267–3.777]
**Navia**	Asturias	8815	3378	51 44.8	1.138 [0.848–1.497]	46 38.86	1.154 [0.845–1.539]	5 4.93	1.013 [0.329–2.365]
**Torrelavega**	Cantabria	53944	3548	261 250.92	1.040 [0.918–1.174]	233 224.54	1.038 [0.909–1.18]	28 26.37	1.062 [0.705–1.534]
**Güeñes**	Bizkaia	5642	3730	32 28.53	1.121 [0.767–1.583]	30 25.60	1.172 [0.791–1.673]	2 2.93	0.682 [0.083–2.465]
**Zalla**	Bizkaia	7629	3730	38 32.97	1.153 [0.816–1.582]	34 29.55	1.151 [0.797–1.608]	4 3.42	1.171 [0.319–2.997]
**Zaragoza**	Zaragoza	592105	2761	2970 2681.70	1.108 [1.068–1.148]	2664 2396	1.112 [1.070–1.155]	306 285.65	1.071 [0.955–1.198]

Observed and expected cases, and standardised mortality ratio.

Inhab: Number of inhabitants. Obs: observed. Exp: expected. SMR: Standardised mortality ratio. CI: Confidence interval

**Figure 1:**
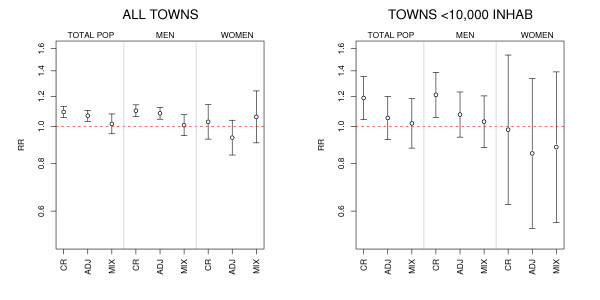
**Relative risks (RR) and 95% confidence intervals for towns situated less than 2 km from paper and board industries, estimated using different models**. CR = crude RRs, ADJ = adjusted RRs (population size, illiteracy, unemployment, farming, income and persons per dwelling), MIX = RRs adjusted for the above variables and including province as a random effects term. INHAB = inhabitants.

Figure 1 depicts the RRs of dying from lung cancer in towns with an EPER-registered industry in the paper, pulp and board sector, estimated on the basis of the various regression models used. Whereas, the RRs yielded by Poisson regression models were generally higher than those obtained from mixed models and displayed statistically significant excess risks among men, the adjusted estimates of the RRs obtained from mixed models showed no statistically significant associations for either sex.

**Figure 2:**
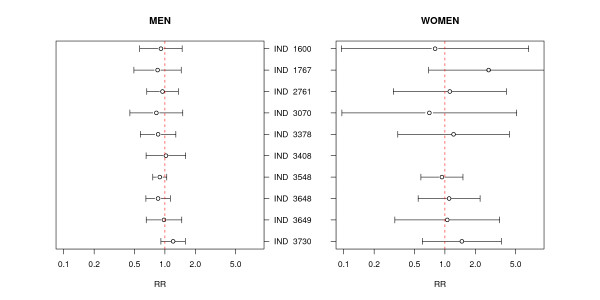
**Relative risks (RR) and 95% confidence intervals for towns situated less than 2 km from 10 paper, pulp and board industries**. Estimated RRs adjusted for population size, illiteracy, unemployment, farming, income and persons per dwelling. IND = industry code. Analysis restricted to a radius of 50 km. For all industrial foci, only towns with fewer than 10,000 inhabitants were considered, with the exception of industries 2761, 3548 and 3648 (in all cases since there were no small towns exposed).

Figure 2 displays the adjusted RR in the environs of each of the industries having a town lying less than 2 km away, with towns having no EPER-registered industry within a 2-kilometre radius being used as reference. Although the results varied widely among sites, most of these failed to register an RR above unity. Highly variable RRs with wide confidence intervals were observed for women, with no statistically significant result in evidence. Only industry 3730 registered an increased RR for men and the total population, which proved statistically significant in the crude but not in the adjusted analysis.

Analysis of risk gradient with distance yielded significant trends solely in industries 2761 and 3730 (Table 3). Small, though statistically significant, rises in risk with proximity to industry were observed in industry 3730 for the overall and male populations, namely, 1.043 (1.006 – 1.082) for the total population, and 1.046 (1.007 – 1.086) for men. Industry 2761 displayed a more consistent association, with increased RRs being recorded for both the total population and men, at most distances. Tests for trend were likewise significant, showing risk increasing as distance to industrial complexes decreased, with figures of 1.290 (1.114 – 1.493) for the overall population and 1.307 (1.124 – 1.520) for men. Attention should be drawn to the excess risk in respect of the town situated in the 5–10 km band, with RRs of 2.411 (1.270 – 4.576) for the total population and 2.621 (1.378 – 4.984) for men. This town has 1042 inhabitants and registered 4.5 expected and 10 observed cases in men. It also displayed excess cases in women, but the RR was considerably attenuated and its statistical significance disappeared when the confounding socio-demographic variables were included in the models (Table 3).

**Table 3:** Relative risks (RR) and 95% confidence intervals (CI) for towns situated at increasing distances from industries 2761 and 3730; and risk gradients with growing proximity to such industries. Adjusted estimates**

Distance		Total towns				Towns with fewer than 10,000 inhabitants		
	
	No*	Total	Men	Women	No*	Total	Men	Women
				
		RR [95% CI]	RR [95% CI]	RR [95% CI]		RR [95% CI]	RR [95% CI]	RR [95% CI]
				Industry 2761				

< 5 km	1	1.437 [0.861–2.397]	1.439 [0.847–2.445]	1.340 [0.174–10.301]	0	-	-	-
5 – 10 km	1	**2.411** **[1.270–4.576]**	**2.621** **[1.378–4.984]**	-	1	**2.425** **[1.291–4.557]**	**2.631** **[1.399–4.948]**	-
10 – 15 km	5	**1.500** **[1.006–2.238]**	**1.525** **[1.010–2.303]**	1.216 [0.228–6.493]	4	**1.503** **[1.009–2.238]**	**1.527** **[1.013–2.302]**	1.220 [0.229–6.483]
15 – 20 km	8	**1.384** **[1.010–1.896]**	1.367 [0.981–1.904]	1.486 [0.536–4.122]	8	**1.391** **[1.032–1.875]**	**1.371** **[1.001–1.879]**	1.500 [0.573–3.927]
20 – 50 km Reference level	79	1	1	1	79	1	1	1

Trend p-value		**0.022**	**0.022**	0.776		**0.0003**	**0.0003**	0.813

Distance				Industry 3730				

< 5 km	4	1.150 [0.899–1.471]	1.157 [0.894–1.496]	1.003 [0.419–2.401]	4	**1.308** **[1.003–1.707]**	**1.344** **[1.017–1.775]**	1.005 [0.401–2.518]
5 – 10 km	3	0.901 [0.666–1.218]	0.873 [0.637–1.196]	1.127 [0.393–3.230]	3	1.118 [0.805–1.554]	1.125 [0.798–1.587]	1.073 [0.349–3.305]
10 – 15 km	14	0.984 [0.873–1.110]	0.969 [0.854–1.099]	1.093 [0.751–1.589]	8	**1.400** **[1.096–1.789]**	**1.429** **[1.104–1.849]**	1.161 [0.525–2.569]
15 – 20 km	18	0.941 [0.862–1.026]	0.931 [0.849–1.022]	0.987 [0.761–1.281]	12	0.950 [0.789–1.143]	0.994 [0.819–1.206]	0.568 [0.284–1.137]
20 – 50 km Reference level	119	1	1	1	108	1	1	1

Trend p-value		0.810	0.938	0.768		**0.045**	**0.038**	

*Number of towns within this distance range. **Adjusted for population size, illiteracy, unemployment, farming, income and persons per dwelling. Statistically significant results shown as bold.

**Figure 3:**
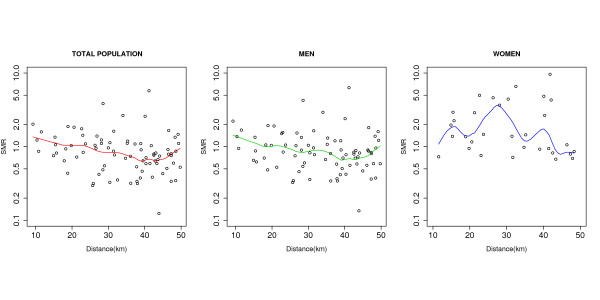
**Towns of < 10,000 inhabitants situated less than 50 km from industry 2761: standardised mortality ratio (SMR) and distance to industry**. Towns with SMR = 0 eliminated to enable gradient in women to be more clearly observed. Semilogarithmic scale. Graph shows total population (82 towns) at left, men (79 towns) in the centre, and women (32 towns) at right. Local weighted scatterplot smoothing (lowess) techniques were applied for plotting the line of trend using a span of 0.3.

Figure 3 graphically depicts the SMRs of towns lying less than 50 kilometres from industry 2761 and their respective distances to it. As distance to this industry decreased, a certain increase in trend was observed for men and the total population, but not for women.

## Discussion

This is one of the first studies to use publicly available EPER information to explore the health effects of industrial pollution emitted by one sector. The results of this study do not support the hypothesis that residence in towns lying very close to paper, pulp and board industries is associated with excess lung cancer mortality. In general, the effect estimators were close to unity and lay at the limit of statistical significance, and there was a marked heterogeneity of effect as between the various installations, something that might indicate that joint analyses are not overly informative. When the installations were studied separately, however, one of them revealed a consistent higher risk of dying from lung cancer due to excess mortality among men; this association was not in evidence among women. A result borderline statistically significant has been found for another focus that have to be interpreted with caution having into account the large number of associations studied that could produce some spurious significant results.

Environmental and industrial pollution has a proven influence on lung cancer incidence and mortality [7,9,12,14]. Occupational studies have shown associations between toxic agents and lung cancer, but the possible association between industries and lung cancer in the general population possibly should be studied by means of "ad hoc" designs. It is likely, moreover, that the effect had on this tumour at population level by isolated environmental exposures deriving from specific industries may be small, thereby rendering detection of possible existing associations difficult. One of the advantages of the design chosen is precisely its high power, resulting from the inclusion of a greater number of subjects. Another of the advantages afforded by this approach is that the analysis can be repeated in future, a feature that is of the utmost importance for the purpose of monitoring and controlling the effects of environmental pollution.

Our study also has important limitations. Working with small areas means that the data evince wide random variability, which particularly affects women. Yet, despite its drawbacks, a small-area study minimises the possible ecological bias associated with the nature of the study *per se*.

One important exposure that could confound the results is smoking. This variable could not be included in the models, since there was no information on it at a municipal level. We sought to minimise this problem by performing a separate analysis by sex and adjusting for socio-demographic variables that could in themselves define subgroups with different proportions of smokers. Nevertheless, this adjustment was only indirect and partial, and there is thus a high likelihood of our results having been influenced by tobacco-related factors. Occupational exposures may also have influenced the difference between men and women, something impossible to control for due to lack of data. Another source of the gender differences in lung cancer could be the interaction of environment air pollutants and smoking, taking into account the low smokers prevalence in women generations before 1940 [51].

In order to attempt to reduce any possible biases deriving from confounding variables not considered in the study, mixed models were fitted with province as the random effects term, something that constitutes a more conservative option, and try to reduce the risk of ecologic bias [52]. A further point to be borne in mind is that some installations for which relative risks of over 1 were observed, are situated in regions with numerous industries emitting into the air. So the industry 2761 has two another EPER industrial plants near, but the industry 3730 does not have any other. This is a factor that may pose a problem when it comes to interpreting the results.

Assuming an isotropic model, this study uses distance to the pollutant source as a proxy of exposure, which in turn introduces a misclassification problem because real exposure is critically dependent on prevailing winds, geographic accidents and emissions into aquifers. Another possible bias stems from using centroids as co-ordinates for positioning a town's entire population, when, in reality, the population may be considerably dispersed and, by extension, misclassified. This classification error becomes much less important in smaller-sized towns, so that analysis of the effects observed when towns of over 10,000 inhabitants are eliminated would serve to confirm or discard the results obtained using all the data. Another contributor to lung cancer incidence are the traffic related emissions [53,54], but our analysis restricted to small tows in which this problem would be less relevant, show similar results.

In 2001, according to the EPER, paper, pulp and board industries reported emitting approximately 4,500 metric tonnes (mt) of carbon monoxide, 1,500,000 mt of carbon dioxide, 2,500 mt of NMVOC (non-methane volatile organic compounds), 5,000 mt of nitrogen dioxide, 7,600 mt of sulphur dioxide and 1,325 mt of PM_10 _particles into the air. These figures evidently do not include information from industries that failed to acknowledge having exceeded the pre-established pollutant thresholds, and correspond to the end of the period under review. The limitations of this work due to the own limitations of the EPER registry have to be taken in consideration. Specially having into account that the reporting has been voluntary till 2007 and the industries still are in the adaptation phase to the regulation. One of the most important consequences is the unknown quality of the emission reported data, as well as the absence of emission historical data -particularly important bearing in mind the latency period of lung cancer-. Although the majority of the industrial sites in this work began their activity in the 50's and 60's, it is in not the case for all of them, and in no case did we have pollution data prior to 2001 or even the date when the respective industries started their activities, something that would be important for evaluation of results. The emission levels for each of the facilities could not have been included as exposure covariate in the regression models, in spite of its potential roll in the differences among the industrial areas.

Exposures deriving from emissions on the part of paper, pulp and board industries involve complex mixtures. Most studies connected with their possible influence on lung cancer pertain to the field of occupational exposure [21-23]. A compound with one of the highest emissions is SO_2_. This, along with SH_2 _and other substances [55], together produce the characteristically pungent odour that impregnates the area surrounding such installations throughout their working lives, and it has been suggested that this pollutant may contribute to pulmonary carcinogenesis [56-58], although probably it could be considered more as an indicator of airborne emissions rather than the suspected carcinogen [59]. Emissions from these types of installations give rise to considerable disorders and symptoms (eye and nasal disorders, and cough) in neighbouring populations [60].

Lastly, mention should be made of the fact that the EPER will soon be replaced by the European Pollutant Release and Transfer Register (E-PRTR), which will include more comprehensive information on industrial pollution from 91 substances and 65 industrial activities, plus information on waste management by such facilities.

## Conclusion

Although no association between paper, pulp and board industries and lung cancer has been shown, it cannot be ruled out. In the environs of two of the industries, risk was observed to increase with proximity. The absence of relevant information and the study's ecological nature renders interpretation of the results in terms of cause and effect difficult. These types of ecological studies, which use distance as an exposure variable, are of great value as exploratory techniques, but their design makes it difficult for some type of association to be found. Nevertheless, were such an association to be found, it should guide research, lending support to the search for more complex forms of analysis.

The study of cancer mortality in areas surrounding pollutant foci is an useful tool for environmental surveillance, and serves to highlight areas of interest susceptible to being investigated by *ad hoc *studies. Despite present limitations, recognition is therefore due to the advance represented by publication of the EPER and the study of pollutant foci.

## Competing interests

The authors declare that they have no competing interests.

## Authors' contributions

SM–C and GL–A conceived the idea, performed the statistical analyses and SM–C drafted the manuscript. NA, BP–G, MP, JG–P and GL–A revised the manuscript for important intellectual content. All authors contributed to the final version of the manuscript.

## Pre-publication history

The pre-publication history for this paper can be accessed here:


